# Blood Count Recovery Following Induction Therapy for Acute Myeloid Leukemia in Children Does Not Predict Survival

**DOI:** 10.3390/cancers14030616

**Published:** 2022-01-26

**Authors:** Lauren Pommert, Todd M. Cooper, Robert B. Gerbing, Lisa Brodersen, Michael Loken, Alan Gamis, Richard Aplenc, Todd A. Alonzo, Edward Anders Kolb

**Affiliations:** 1 Division of Oncology, Cancer and Blood Diseases Institute, Cincinnati Children’s Hospital Medical Center, Cincinnati, OH 45229, USA; lauren.pommert@cchmc.org; 2 Department of Pediatrics, University of Cincinnati College of Medicine, Cincinnati, OH 45229, USA; 3 Division of Hematology/Oncology, Cancer and Blood Disorders Center, Seattle Children’s Hospital, Seattle, WA 98105, USA; 4 Children’s Oncology Group, Monrovia, CA 91016, USA; rgerbing@childrensoncologygroup.org; 5 Hematologics Inc., Seattle, WA 98121, USA; Lisa@hematologics.com (L.B.); mrloken@hematologics.com (M.L.); 6 Children’s Mercy Hospital and Clinics, Kansas City, MO 64108, USA; agamis@cmh.edu; 7 Division of Oncology, The Children’s Hospital of Philadelphia, Philadelphia, PA 19104, USA; aplenc@chop.edu; 8 Department of Population and Public Health Sciences, University of Southern California, Los Angeles, CA 90032, USA; talonzo@childrensoncologygroup.org; 9 Nemours Center for Cancer and Blood Disorders/Nemours Children’s Health, Wilmington, DE 19803, USA; Edward.Kolb@nemours.org

**Keywords:** pediatric acute myeloid leukemia, childhood acute myeloid leukemia, clinical trial response assessment, IWG criteria

## Abstract

**Simple Summary:**

International Working Group (IWG) and European LeukemiaNet (ELN) adult response definitions are currently used to evaluate the efficacy of new agents for childhood acute myeloid leukemia (AML); however, the criteria are not consistent with consensus definitions used in pediatric trials or the common practice of intensifying treatment prior to full hematopoietic recovery of ANC ≥ 1000 cells/μL and platelets ≥ 100 cells/μL. This retrospective analysis of the two most recent Phase 3 AML trials in the Children’s Oncology Group assesses the incidence, timing, and prognostic significance of count recovery following induction chemotherapy in children with AML. These data confirm that awaiting count recovery to meet adult criteria does not reflect standard practice in pediatric AML and IWG/ELN-defined CR does not have a significant impact on survival in children. Continuing to use adult IWG/ELN count recovery definitions limits childhood AML drug development by underestimating response, and therefore, updated response criteria are needed for pediatric AML patients.

**Abstract:**

International Working Group (IWG) and European LeukemiaNet (ELN) response definitions are utilized to evaluate the efficacy of new agents for childhood acute myeloid leukemia (AML) for regulatory purposes. However, these criteria are not consistent with definitions used in pediatric AML trials or with standard pediatric practice to proceed with subsequent therapy cycles prior to IWG/ELN-defined count recovery. We retrospectively analyzed data from the two most recent Phase 3 pediatric AML clinical trials conducted by the Children’s Oncology Group (COG) to assess the incidence, timing, and prognostic significance of count recovery following induction chemotherapy. Of the patients with fewer than 5% bone marrow blasts at the end of first induction, 21.5% of patients proceeded to a second induction cycle prior to achieving ANC ≥ 500 cells/μL and platelets ≥ 50,000 cells/μL, both well below the IWG/ELN thresholds of ANC > 1000 cells/μL and platelets > 100,000 cells/μL. In these two sequential childhood AML Phase 3 trials, neither ANC nor platelet recovery predicted survival. Intensification of treatment through the initiation of subsequent therapy cycles prior to attainment of IWG/ELN-defined CR is common practice in clinical trials for children with AML, suggesting that updated response definitions are needed for pediatric AML.

## 1. Introduction

Acute myeloid leukemia (AML) in children and adults represent a phenotypically heterogeneous and genetically complex subtype of hematopoietic malignancies. There are approximately 20,000 newly diagnosed cases of AML in the United States each year with an average age at diagnosis of 68 years; however, fewer than 500 of these cases occur in children under the age of 15. Given this differential age distribution [1], clinicians have long assumed that AML observed in older adults is distinct from that seen in children. Age is not a defining characteristic of AML according to the World Health Organization (WHO); rather, the category of AML with recurrent genetic abnormalities captures many of the structural variants seen in more than 50% of children with AML and fewer than 15% of older adults [2,3,4]. Assessment of treatment response for children and adults with AML in the United States is currently based on the International Working Group (IWG) criteria, first published in 1990 [5] and updated in 2003 [6]. The IWG criteria require a peripheral complete blood count (CBC) and histologic quantification of bone marrow blasts by microscopy, and define complete response (CR) as fewer than 5% bone marrow blasts with an absolute neutrophil count (ANC) > 1000 cells/μL and platelets (plt) > 100,000 cells/μL. Similar criteria are followed by the European LeukemiaNet (ELN) in adults with AML [7]. These response criteria have remained the standard by which the efficacy of new drugs is measured in clinical trials in both adults and children. 

In 2010, Walter et al. demonstrated clearly that in adult AML patients treated 15 to 35 years ago, it was routine to await count recovery at the end of induction to evaluate for residual dysplasia [8]. During this 19-year study period, 97% of adult patients with less than 5% blasts had platelet count recovery to greater than 100,000 cells/μL after initial induction therapy, and count recovery was associated with improved survival. These data support the IWG and ELN response criteria for AML in adults. Recognizing that these criteria have never been validated in children, herein we assess the incidence of count recovery and its prognostic impact on survival in children with AML treated in the most recent AML trials from the Children’s Oncology Group (COG). We hypothesized that the adult IWG/ELN criteria do not predict survival in pediatric patients. 

## 2. Materials and Methods

### 2.1. Patients

**Figure 1:**
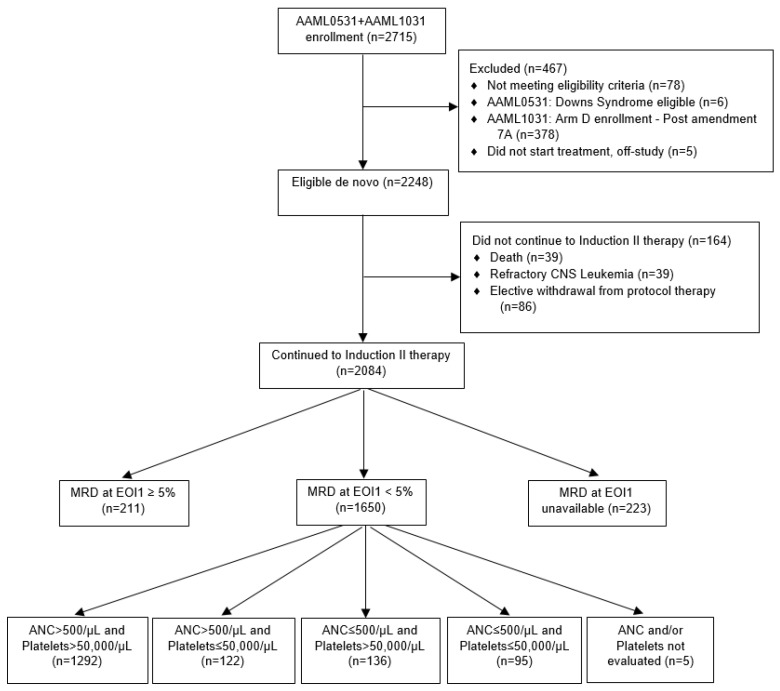
CONSORT diagram.

We retrospectively analyzed data from the two most recent Phase 3 pediatric clinical trials for de novo AML conducted by the COG in the United States. The eligibility, therapy, and results for these trials have been previously reported [9,10]. The CONSORT diagrams describing patients analyzed in this dataset are included in Figure 1. Data from the COG cohort include more than 2700 patients 1–29 years of age with AML diagnosed between 2006 and 2018 and enrolled in AAML0531 (NCT00372593) [9] and AAML1031 (NCT01371981) [10]. Patients with Down syndrome AML (DS-AML) in AAML0531 (*n* = 6) were excluded from this analysis, as were all patients enrolled in AAML1031’s Arm D expansion cohort (*n* = 378) because response data were not collected for these patients. Patients who withdrew consent or went off-study for other reasons were censored at that date. 

Among patients enrolled in both trials, 1861 (89.3%) had RD assessed by centrally performed difference-from-normal (ΔN) flow cytometry at the end of induction I, which was previously shown to be superior to morphology in assessing response in children [11]. Those with <5% RD by ΔN (*n* = 1645) achieved a complete response and were evaluated for time to and incidence of count recovery. Comprehensive CBC data were not collected for each patient. Instead, investigators were required to report whether patients achieved peripheral ANC of 500 cells/μL and non-transfused platelet count of 50,000 cells/μL prior to Induction II, and the date each parameter was achieved. Both COG trials recommended but did not require that Induction II begin when ANC > 1000 cells/μL and platelets > 75,000 cells/μL (Table 1). Survival analysis based on count recovery group was performed using disease-free survival (DFS) and overall survival (OS) from end of induction I, and excluded the 60 patients in AAML1031 (Arm C) who received sorafenib for HAR FLT3-ITD disease (*n* = 1585; Figure 1).

### 2.2. Statistical Analysis

Statistical analyses were performed with SAS version 9.4 (SAS Institute Inc., Cary, NC, USA). The Kaplan–Meier method was applied to estimate probabilities of survival with standard errors according to Greenwood and compared with the log-rank test [12]. Survival curves were compared using the log-rank test and based on count recovery (ANC ≥ 500 cells/μL only, platelets ≥ 50,000 cells/μL only, both ANC ≥ 500 cells/μL and platelets ≥ 50,000 cells/μL, and neither ANC ≥ 500 cells/μL nor platelets ≥ 50,000 cells/μL). OS was calculated from end of induction I to death of any cause, and DFS was defined as time from end of induction I to treatment failure, relapse, secondary malignancy, or death. Cumulative incidence functions of ANC/platelet recovery, relapse, or early death were constructed according to Kalbfleisch and Prentice [13]. The Cox proportional hazards model was used for multivariable analysis of outcomes [14]. For consistency in multivariable survival analysis, COG risk groups for this analysis were defined by current COG Phase 3 (AAML1831, NCT04293562) cytomolecular risk stratification and MRD [15] rather than original study-assigned risk group. Proportions were compared between groups using the chi-square test or Fisher’s exact test when data were sparse. *p* values <0.05 were considered significant. Living patients were censored at date of last follow-up. Data were frozen at 31 December 2019.

## 3. Results

### 3.1. Proportion of Patients with Count Recovery

Among all patients with fewer than 5% bone marrow AML blasts by centralized ΔN flow cytometry (*n* = 1645), the proportion of patients who proceeded to Induction II prior to recovery of ANC ≥ 500 cells/μL and platelet count ≥ 50,000 cells/μL was 21.5% (7.4% with ANC only + 8.3% with platelets only + 5.8% with neither ANC nor platelet recovery; Table 2). 

**Figure 2:**
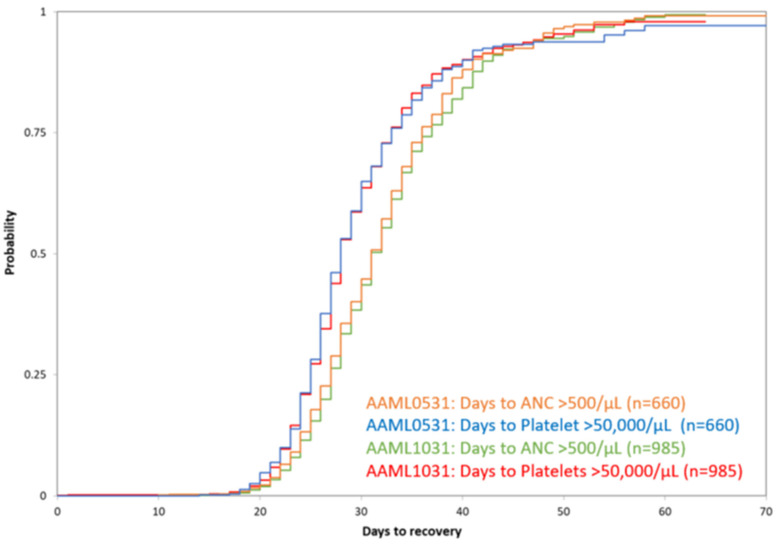
Cumulative incidence of count recovery. Count recovery in all patients with ≤5% bone marrow blasts by central ΔN flow cytometry at the end of Induction I who went on to receive Induction II therapy on AAML0531 and AAML1031.

In a cumulative incidence of count recovery analysis, 96.2% and 98.1% of patients recovered ANC ≥ 500 cells/μL or platelet counts ≥ 50,000 cells/μL across the two Phase 3 COG trials at 42 and 49 days from the start of induction therapy, respectively. Only 86.3% and 92.4% reported recovery of both ANC ≥ 500 cells/μL and platelets ≥ 50,000 cells/μL at 42 and 49 days from the start of induction therapy, respectively (Figure 2). Additional patient characteristics by count recovery group can be found in Supplemental Table S1. 

### 3.2. Survival According to Count Recovery 

**Figure 3:**
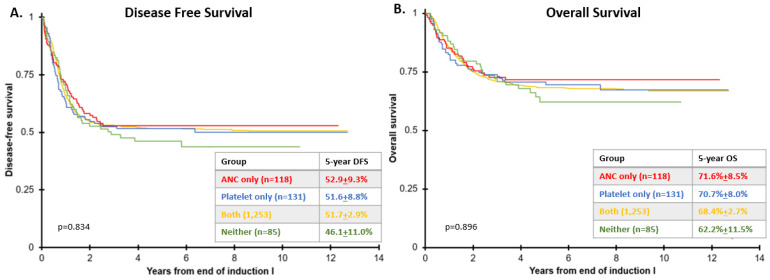
Survival by count recovery from end of induction I (EOI1). (**A**) Disease-free survival (DFS) and (**B**) overall survival (OS) from EOI1 are reported according to count recovery parameters in patients with <5% bone marrow blasts by central ΔN flow cytometry who received Induction I and II therapy in AAML0531 and AAML1031.

Survival analysis based on count recovery group was performed among patients with fewer than 5% bone marrow AML blasts by centralized ΔN flow cytometry, and is reported in Figure 3. Median time to follow up for all patients alive at last contact was 4.7 years (range 0.2–7.6 years). Among responders, there was no significant association between count recovery parameters and five-year DFS (*p* = 0.843) or OS (*p* = 0.896) (Figure 3) or RR (Supplemental Table S2). To address the trend of inferior outcomes for patients who recovered neither ANC nor platelets (Figure 3, green group), we compared that group to all other patients (Figure 3, red, blue and yellow groups). In that comparison, there was no significant difference in either five-year DFS (46.1% ± 11.0% vs. 51.8% ± 2.6%, respectively; *p* = 0.453) or OS (62.2% ± 11.5% vs. 68.8% ± 2.5%, respectively; *p* = 0.596). There was a trend toward decreased outcomes in the multivariable Cox analysis, as shown in Supplemental Tables S2 and S3. Variables which independently predicted outcomes included: sorafenib exposure for HAR FLT3-ITD patients in AAML1031, Gemtuzumab exposure in AAML0531, risk group stratification, WBC count at diagnosis, and HSCT in CR1 (Supplemental Table S2). An analysis comparing the individual treatment arms in each study also did not demonstrate that count recovery was predictive of EFS or OS (Supplemental Table S3).

## 4. Discussion

### 4.1. Waiting for Count Recovery per IWG/ELN Criteria for CR Does Not Reflect Standard Practice in Pediatric AML

The standard AML treatment approach over the past 40 years for both children and adults has been anthracyclines combined with cytarabine, though the therapeutic landscape is now evolving to include more targeted therapies. We now have a better understanding of AML biology, response, risk classification, and the impact of each on survival. Accordingly, as we evaluate therapies for children, we must define relevant response criteria in childhood AML to support the development of promising new therapies to advance cures. Delays in count recovery in adults with AML are thought to reflect the persistence of leukemia, often with a myelodysplasia (MDS) phenotype. Childhood AML often has more aggressive biology compared to that in adults and the MDS phenotype is quite rare [3,4]. Awaiting marrow recovery to IWG/ELN defined count thresholds has not been the recommended practice in pediatric AML Phase 3 trials. Pediatric patients often start subsequent chemotherapy cycles prior to count recovery to IWG/ELN parameters (Table 1) because dose timing and intensification has demonstrated improved outcomes in both North American and European trials for de novo pediatric AML [16,17]. In the most recent completed COG Phase 3 trials, 21.5% of patients proceeded to a second cycle of induction therapy prior to recovering ANC > 500 cells/μL and/or platelets > 50,000 cells/μL (Table 1), which are well below the IWG/ELN criteria of ANC > 1000 cells/uL and platelets > 100,000 cells/uL. The current COG de novo AML Phase 3 study, AAML1831 (NCT0429356), recommends that subsequent cycles of therapy should begin when ANC > 500 cell/uL and platelets > 50,000 cells/uL (Table 1). The AAML1831 study committee implemented these recommendations based on observations from AAML0531/1031 that investigators were not waiting for count recovery to suggested parameters (personal communication). Contrary to the findings of Walter et al., the data from AAML0531/1031 confirm that a significant proportion of pediatric patients do not reach the IWG/ELN thresholds for recovery before the start of subsequent therapeutic cycles and highlight the prioritization of therapy intensification by pediatric oncologists over complete hematopoietic regeneration.

### 4.2. Count Recovery Does Not Significantly Impact Survival in These Large Pediatric Datasets

The current IWG/ELN criteria for response imply that the absence of full hematopoietic recovery (ANC > 1000 cells/uL and plt > 100 cells/uL) is prognostic in adults with AML. Although retrospective studies have suggested inferior outcomes for adult patients with CRi/CRp compared to those with CR [8,18], this has never been validated in pediatric AML patients. While our pediatric cohort is not adequately powered to specifically answer this survival question, it represents the largest US dataset available to review incidence and survival associated with count recovery, and the sample size is comparable to the Walter cohort [8]. Our retrospective evaluation of the available COG de novo AML data was unable to detect a significant difference in DFS and OS based on ANC and platelet recovery in children (Figure 3). Likewise, recent BFM data also confirm that count recovery does not predict survival in the setting of first relapse of AML [19,20]. Although in Figure 3 the DFS and OS appear lower in the COG cohort without ANC or platelet recovery by the end of induction I, this trend was not statistically significant. We do note that the number of patients with EOI1 MRD positivity, a known prognostic factor, was higher in the cohort without ANC and platelet recovery when compared to other cohorts (*p* = 0.063) (Supplemental Table S1). Survival in all COG cohort groups was comparable until two years of follow-up, after which six patients in AAML0531 experienced late relapses. MRD rates were similar between these late relapse patients and the rest of the cohorts. 

One of the limitations of this study is the lack of detailed end-of-induction CBC data for each patient enrolled in these clinical trials. Therefore, we are unable to directly refute the IWG/ELN CR criteria (ANC ≥ 1000 cells/µL and platelets ≥ 100,000 cells/µL). As described above, it is not common practice for physicians treating childhood AML to wait for count recovery to these thresholds, and therefore, cut-offs of ANC ≥ 500 of cells/µL and platelets ≥ 50,000 cells/µL were used for data analysis. Data from our colleagues in the International Berlin-Frankfurt-Munster (i-BFM) group confirm this practice, demonstrating that only one-third of patients achieve recovery to IWG/ELN criteria after AML induction (personal communication, Dirk Rheinhardt). It is possible that strict adherence to waiting for count recovery to the IWG/ELN criteria might show more significant survival differences among these groups. 

### 4.3. Cheson Criteria Should Be Reconsidered as the Standard Response Evaluation in Children with AML, and Perhaps Also in Adults

The current IWG and ELN criteria were based upon retrospective data in adults supporting prolonged survival for patients who achieve CR [8]. Specifically, Walter et al. demonstrated that among 1321 patients treated between 1984 and 2004, CR was independently associated with a longer relapse-free survival relative to CRp [8]. In comparison to the COG experience, the Walter et al. data demonstrate clear differences in tolerance for waiting for full count recovery that may be influenced by the underlying biology of adult AML compared to childhood AML. Despite this, CR by IWG/ELN criteria are the standard applied by regulatory authorities to new drug approvals in children. The Food and Drug Administration goes so far as to define a treatment failure as a failure to achieve CR by IWG criteria [21]. As we consider how best to assess response in children with AML, we must consider that the standards of care for children and adults captured in this dataset are clearly and substantially different. Therefore, using the IWG/ELN criteria risks an underestimate of response in children, even if CRp is included in the overall response assessment. 

Although CR per IWG and ELN criteria has historically been used as a surrogate for survival in adults, recent studies question the reliable association between CR and survival, suggesting it may be time to reconsider these definitions for response evaluation in both adult and pediatric AML [22]. Multiple recent adult studies have demonstrated significant improvements in CR rates without improvement in survival [23,24], inferior CR rates with comparable survival [25,26], and absence of CR with improved survival [27]. These data suggest that response, as currently defined by the IWG/ELN, may over- or underestimate survival in adults with AML treated with contemporary therapies.

## 5. Conclusions

In the largest available US pediatric cohort, we are unable to confirm an association between peripheral blood count recovery and survival, despite a comparable sample size to the Walter et al. cohort. Continuing to use adult IWG/ELN response assessment definitions places severe limitations on childhood AML drug development by classifying lack of CR using these guidelines as treatment failure. Additionally, these definitions are not aligned with the standard of care for intensification of pediatric AML therapy and are not followed by pediatric oncologists.

Given the data presented here, response criteria must be reconsidered for pediatric patients with AML. International cooperative groups are currently working to compile new, standard definitions for both count recovery thresholds and remission status in order to more accurately define treatment responses in future pediatric AML trials. In the meantime, we propose that CR, CRp and CRi are all valid primary study endpoints.

## Figures and Tables

**Table 1:** Count recommendations for proceeding with the next cycle of chemotherapy.

	COG De Novo Cohort (AAML0531 and AAML1031 *)	Current COG AML Study (AAML1831 *)	IWG/ELN
ANC	>1000 cells/μL	>500 cells/μL	>1000 cells/μL
Platelets	>75,000 cells/μL	>50,000 cells/μL	>100,000 cells/μL

Table Legend: * AAML0531 (NCT00372593), AAML1031 (NCT01371981), AAML1831 (NCT04293562); Abbreviations: AML, acute myeloid leukemia; ANC, absolute neutrophil count; COG, Children’s Oncology Group; ELN, European LeukemiaNet; IWG, International Working Group.

**Table 2:** Count recovery following first induction on AAML0531 and AAML1031.

	Patients with <5% Marrow Disease by ΔN Flow Cytometry	ANC Threshold Only (>500/μL)	Platelet Threshold Only (>50,000/μL)	Met Both (ANC > 500/μL and Plt > 50,000/μL)	Met Neither (ANC ≤ 500/μL and Plt ≤ 50,000/μL)	ANC and/or Platelets Not Evaluated during Reporting Period
AAML0531	660	57 (8.6%)	55 (8.3%)	507 (76.8%)	41 (6.2%)	4 (0.6%)
AAML1031	985	65 (6.6%)	81 (8.2%)	785 (79.7%)	54 (5.5%)	1 (0.1%)
Combined	1645	122 (7.4%)	136 (8.3%)	1292 (78.5%)	95 (5.8%)	5 (0.3%)

Table abbreviations: ANC, absolute neutrophil count; ΔN, difference from normal; Plt, platelet.

## Data Availability

The Children’s Oncology Group Data Sharing policy describes the release and use of COG individual subject data for use in research projects in accordance with National Clinical Trials Network (NCTN) Program and NCI Community Oncology Research Program (NCORP) Guidelines. Only data expressly released from the oversight of the relevant COG Data and Safety Monitoring Committee (DSMC) are available to be shared. Data sharing will ordinarily be considered only after the primary study manuscript is accepted for publication. For phase 3 studies, individual-level de-identified datasets that would be sufficient to reproduce results provided in a publication containing the primary study analysis can be requested from the NCTN/NCORP Data Archive at https://nctn-data-archive.nci.nih.gov/, accessed on 22 November 2021. Data are available to researchers who wish to analyze the data in secondary studies to enhance the public health benefit of the original work and agree to the terms and conditions of use. For non-phase 3 studies, data are available following the primary publication. An individual-level de-identified dataset containing the variables analyzed in the primary results paper can be expected to be available upon request. Requests for access to COG protocol research data should be sent to: datarequest@childrensoncologygroup.org. Data are available to researchers whose proposed analysis is found by COG to be feasible and of scientific merit and who agree to the terms and conditions of use. For all requests, no other study documents, including the protocol, will be made available and no end date exists for requests. In addition to above, release of data collected in a clinical trial conducted under a binding collaborative agreement between COG or the NCI Cancer Therapy Evaluation Program (CTEP) and a pharmaceutical/biotechnology company must comply with the data sharing terms of the binding collaborative/contractual agreement and must receive the proper approvals.

## Supplementary Materials

The following supporting information can be downloaded at: https://www.mdpi.com/article/10.3390/cancers14030616/s1, Table S1. Patient characteristics by count recovery. Table S2. Multivariable analysis for overall survival (OS), disease free survival (DFS), and relapse risk (RR) from end of induction I. Table S3. Multivariable analysis for overall survival (OS), disease free survival (DFS), and relapse risk (RR) from end of induction I by study treatment arm.
