# Supplementary material for: Blood Count Recovery Following Induction Therapy for Acute Myeloid Leukemia in Children Does Not Predict Survival
Source: Cancers (Basel). 2022 Jan 26;14(3):616. doi: 10.3390/cancers14030616 (PMC8833679; doi:10.3390/cancers14030616)
Supplement: Supplementary file 1 [file cancers-14-00616-s001.zip › cancers-1498712-supplementary.pdf]

Table S1: Patient characteristics by count recovery

	All		MRD<5%, ANC>500/µL, Platelets>50,000/µL		MRD<5%, ANC>500/µL, Platelets≤50,000/µL		MRD<5%, ANC≤500/µL, Platelets>50,000/µL		MRD<5%, ANC≤500/µL, Platelets≤50,000/µL		
**Characteristic**	**N**	**%**	**N**	**%**	**N**	**%**	**N**	**%**	**N**	**%**	**p**
**Total**	1645		1292		122		136		95		
**Study**											
**AAML0531**	660	40.1%	507	39.2%	57	46.7%	55	40.4%	41	43.2%	0.392
**Treatment Arm**											
**Arm A (No GO)**	326	49.4%	241	47.5%	31	54.4%	32	58.2%	22	53.7%	0.352
**Arm B (GO)**	334	50.6%	266	52.5%	26	45.6%	23	41.8%	19	46.3%	
**AAML1031**	985	59.9%	785	60.8%	65	53.3%	81	59.6%	54	56.8%	
**Treatment Arm**											
**Arm A (No Bortezomib)**	453	46.0%	351	44.7%	33	50.8%	44	54.3%	25	46.3%	0.337
**Arm B (Bortezomib)**	472	47.9%	395	50.3%	26	40.0%	32	39.5%	19	35.2%	0.026
**Arm C (Sorafenib)**	60	6.1%	39	5.0%	6	9.2%	5	6.2%	10	18.5%	0.001
**Gender**											
**Male**	831	50.5%	663	51.3%	52	42.6%	65	47.8%	51	53.7%	0.245
**Female**	814	49.5%	629	48.7%	70	57.4%	71	52.2%	44	46.3%	
**Age**											
**0-1 yrs old**	345	21.0%	271	21.0%	25	20.5%	37	27.2%	12	12.6%	0.066
**2-10 yrs old**	568	34.5%	427	33.0%	39	32.0%	60	44.1%	42	44.2%	**0.011**
**≥11 yrs old**	732	44.5%	594	46.0%	58	47.5%	39	28.7%	41	43.2%	0.002
**WBC**											

**<100,00/μL**	1359	82.7%	1065	82.4%	97	79.5%	119	88.1%	78	82.1%	0.290
**≥100,00/μL**	285	17.3%	227	17.6%	25	20.5%	16	11.9%	17	17.9%	
**Race**											
**Non-African American**	1292	87.8%	1011	87.6%	99	90.8%	114	89.1%	68	85.0%	0.626
**African American**	179	12.2%	143	12.4%	10	9.2%	14	10.9%	12	15.0%	
**MRD at EOI1**											
**Negative**	1340	81.5%	1065	82.4%	97	79.5%	110	80.9%	68	71.6%	0.063
**Positive**	305	20.7%	227	19.7%	25	22.9%	26	20.3%	27	33.8%	
**New Risk Group***											
**Low Risk 1**	467	27.7%	360	27.9%	41	33.6%	42	30.9%	24	25.3%	0.443
**Low Risk 2**	667	39.6%	547	42.3%	35	28.7%	52	38.2%	33	34.7%	0.015
**High**	551	32.7%	385	29.8%	46	37.7%	42	30.9%	38	40.0%	0.071
**Age**											
**Median (range)**	9.4	(0 - 29.5)	9.8	(0.02 - 29.5)	9.6	(0 - 26.3)	6.1	(0.2 - 22.2)	10.4	(0.6 - 19.6)	0.001
**WBC x10^3 μL**											
**Median (range)**	19.9	(0.2 - 918.5)	19.9	(0.2 - 918.5)	22.8	(0.9 - 446)	16.7	(1.5 - 550)	24.2	(1.1 - 400)	0.252
**Bone Marrow Blasts %**											
**Median (range)**	67	(0 - 100)	67	(0 - 100)	65	(3.6 - 97)	67.5	(2.8 - 98)	68	(3 - 99)	0.778
**Peripheral blasts (%)**											
**Median (range)**	35	(0 - 100)	35	(0 - 99)	33	(0 - 98)	30	(0 - 98)	46	(0 - 100)	0.254

*Based on risk group definition in AAML1831 (using FLT3/ITD, FLT3/AM, mutations, cytogenetics and MRD). ANC, absolute neutrophil count; EOI1, End of induction I; GO, Gemtuzumab ozogamicin; MRD, minimal residual disease; WBC, white blood cells

Table S2: Multivariable analysis for overall survival (OS), disease free survival (DFS), and relapse risk (RR) from end of induction I

	OS from EOI1				DFS from EOI1			RR from EOI1		
	**N**	**HR**	**95% Cl**	**p**	**HR**	**95% CI**	**p**	**HR**	**95% CI**	**p**
**ANC/PLT recovery groups^**										
**Both platelet and ANC recovery (CR)**	1154	1			1			1		
**Platelet recovery only (≥50 cells/uL)**	127	1.14	0.83 - 1.58	0.421	1.27	0.98 - 1.65	0.073	1.24	0.93 - 1.67	0.150
**ANC recovery only (>500 cells/uL; CRp)**	109	0.89	0.60 - 1.31	0.545	0.92	0.68 - 1.25	0.586	0.93	0.66 - 1.30	0.655
**Neither platelet nor ANC recovery**	80	1.06	0.72 - 1.55	0.766	1.14	0.83 - 1.55	0.418	1.14	0.82 - 1.58	0.443
**Study/Treatment Arm**										
**AAML0531 Arm A or AAML1031 Arms A/B**	1108	1			1			1		
**AAML0531 Arm B (GO)**	305	0.81	0.65 - 1.03	0.081	0.80	0.66 - 0.97	**0.021**	0.74	0.60 - 0.91	**0.005**
**AAML1031 Arm C (Sorafenib)**	57	0.57	0.34 - 0.97	**0.040**	0.45	0.28 - 0.72	**0.001**	0.31	0.18 - 0.55	**<0.001**
**WBC Count**										
**<100,00/µL**	1207	1			1			1		
**≥100,00/µL**	263	1.26	0.99 - 1.60	0.056	1.42	1.18 - 1.72	**<0.001**	1.55	1.25 - 1.92	**<0.001**
**Race**										
**Non-African American**	1291	1			1			1		
**African American**	179	1.31	1.00 - 1.72	0.051	1.05	0.84 - 1.33	0.655	0.96	0.75 - 1.24	0.757
**New Risk Group***										
**Low Risk 1**	413	1			1			1		
**Low Risk 2**	592	2.64	1.90 - 3.68	**<0.001**	2.03	1.62 - 2.54	**<0.001**	2.31	1.82 - 2.93	**<0.001**
**High**	465	6.88	4.95 - 9.55	**<0.001**	4.33	3.42 - 5.47	**<0.001**	4.86	3.78 - 6.24	**<0.001**
**HSCT in CRŦ**	234	0.85	0.65 - 1.11	0.233	0.70	0.55 - 0.90	**0.005**	0.52	0.39 - 0.69	**<0.001**

ANC, absolute neutrophil count; CI, confidence interval; CR, complete response; CRp, complete response with incomplete platelet recovery; DFS, disease free survival; EOI1, end of induction 1; HR, hazard ratio; OS, overall survival; PLT, platelet; RR, relapse risk; HSCT, hematopoietic stem cell transplant; WBC, white blood cell

^CR defined as <5% residual marrow disease by flow cytometry, peripheral ANC of ≥500 cells/μL and a platelet count of ≥50,000 cells/μL for 7 days without transfusion

*Based on risk group definition in AAML1831 (using FLT3/ITD, FLT3/AM, mutations, cytogenetics and MRD)

ŦTime dependent variable

n=175 patients are unknown for either WBC or Race and are excluded from MV analyses above

n=51 patients on AAML1031 have unknown SCT in CR status therefore they are censored at the start time of follow-up period when SCT was received

Table S3: Multivariable analysis for overall survival (OS), disease free survival (DFS), and relapse risk (RR) from end of induction I by study treatment arm

	**OS from EOI1**				**DFS from EOI1**			**RR from EOI1**		
	N	HR	95% Cl	p	HR	95% CI	p	HR	95% CI	p
**AAML0531 Arm A or AAML1031 Arms A/B only**										
**ANC/PLT recovery groups^**										
**Both platelet and ANC recovery (CR)**	870	1			1			1		
**Platelet recovery only (≥50 cells/uL)**	100	1.18	0.81 - 1.71	0.398	1.35	1.01 - 1.82	0.043	1.27	0.91 - 1.78	0.165
**ANC recovery only (>500 cells/uL; CRp)**	82	0.89	0.57 - 1.38	0.595	0.88	0.62 - 1.25	0.476	0.86	0.58 - 1.27	0.439
**Neither platelet nor ANC recovery**	56	0.81	0.50 - 1.33	0.413	1.05	0.72 - 1.52	0.806	1.09	0.74 - 1.58	0.671
**AAML0531 Arm B (GO) only**										
**ANC/PLT recovery groups^**										
**Both platelet and ANC recovery (CR)**	246	1			1			1		
**Platelet recovery only (≥50 cells/uL)**	22	1.13	0.54 - 2.37	0.741	0.98	0.51 - 1.88	0.946	0.95	0.48 - 1.88	0.877
**ANC recovery only (>500 cells/uL; CRp)**	22	1.05	0.45 - 2.42	0.917	1.30	0.70 - 2.44	0.409	1.45	0.75 - 2.78	0.266
**Neither platelet nor ANC recovery**	15	1.18	0.51 - 2.75	0.694	0.99	0.48 - 2.06	0.988	0.99	0.45 - 2.21	0.988

ANC, absolute neutrophil count; CI, confidence interval; CR, complete response; CRp, complete response with incomplete platelet recovery; DFS, disease free survival; EOI1, end of induction 1; HR, hazard ratio; OS, overall survival; PLT, platelet; RR, relapse risk

^CR defined as <5% residual marrow disease by flow cytometry, peripheral ANC of ≥500 cells/μL and a platelet count of ≥50,000 cells/μL for 7 days without transfusion
